# Exploring the origins of EEG motion artefacts during simultaneous fMRI acquisition: Implications for motion artefact correction

**DOI:** 10.1016/j.neuroimage.2018.02.034

**Published:** 2018-06

**Authors:** Glyn S. Spencer, James A. Smith, Muhammad E.H. Chowdhury, Richard Bowtell, Karen J. Mullinger

**Affiliations:** a Sir Peter Mansfield Imaging Centre, School of Physics and Astronomy, University of Nottingham, University Park, Nottingham, NG7 2RD, UK; b Department of Physics, Loughborough University, Leicestershire, LE11 3TU, UK; c Department of Electrical Engineering, College of Engineering, Qatar University, P. O. Box 2713, Doha, Qatar; d Centre for Human Brain Health, School of Psychology, University of Birmingham, Birmingham, B15 2TT, UK

**Keywords:** Simultaneous EEG-fMRI, Artefact correction, Motion artefacts, Motion characterisation

## Abstract

Motion artefacts (MAs) are induced within EEG data collected simultaneously with fMRI when the subject's head rotates relative to the magnetic field. The effects of these artefacts have generally been ameliorated by removing periods of data during which large artefact voltages appear in the EEG traces. However, even when combined with other standard post-processing methods, this strategy does not remove smaller MAs which can dominate the neuronal signals of interest. A number of methods are therefore being developed to characterise the MA by measuring reference signals and then using these in artefact correction. These methods generally assume that the head and EEG cap, plus any attached sensors, form a rigid body which can be characterised by a standard set of six motion parameters. Here we investigate the motion of the head/EEG cap system to provide a better understanding of MAs. We focus on the reference layer artefact subtraction (RLAS) approach, as this allows measurement of a separate reference signal for each electrode that is being used to measure brain activity.

Through a series of experiments on phantoms and subjects, we find that movement of the EEG cap relative to the phantom and skin on the forehead is relatively small and that this non-rigid body movement does not appear to cause considerable discrepancy in artefacts between the scalp and reference signals. However, differences in the amplitude of these signals is observed which may be due to differences in geometry of the system from which the reference signals are measured compared with the brain signals. In addition, we find that there is non-rigid body movement of the skull and skin which produces an additional MA component for a head shake, which is not present for a head nod. This results in a large discrepancy in the amplitude and temporal profile of the MA measured on the scalp and reference layer, reducing the efficacy of MA correction based on the reference signals.

Together our data suggest that the efficacy of the correction of MA using any reference-based system is likely to differ for different types of head movement with head shake being the hardest to correct. This provides new information to inform the development of hardware and post-processing methods for removing MAs from EEG data acquired simultaneously with fMRI data.

## Introduction

Simultaneous EEG-fMRI is a valuable multi-modal technique for investigating brain function. It has been used to study the coupling of trial-by-trial fluctuations between EEG and fMRI signals (Becker et al., 2011; Debener et al., 2005; Mullinger et al., 2013a) and to investigate haemodynamic correlates of brain oscillations (de Munck et al., 2009; Goldman et al., 2002; Laufs et al., 2003). Simultaneous EEG-fMRI has also shown promise in the study of epileptic activity (Moeller et al., 2013; Carney and Jackson, 2014; Kay and Szaflarski, 2014; Centeno and Carmichael, 2014; Gotman et al., 2006) and has potential clinical applications in the diagnosis and treatment of epilepsy (Gotman et al., 2004; Pittau et al., 2014a, 2014b, 2012).

However, the quality of EEG recordings acquired during an fMRI experiment can be compromised by large artefacts due to interaction of the EEG system (EEG cap, cables and amplifier) and head with the magnetic fields used in MRI. These artefacts are grouped into three distinct types: the gradient artefact (GA) (Yan et al., 2009), the pulse artefact (Debener et al., 2008; Ives et al., 1993; LeVan et al., 2013; Mullinger et al., 2013b; Muri et al., 1998; Yan et al., 2010) and the motion artefact (Fellner et al., 2016; Jansen et al., 2012). The motion artefact (MA) results from the movement of the conductive paths formed by the EEG system and the subject's head in the strong static magnetic field of the MRI scanner. Due to the random occurrence and the innate spatial and temporal variability of the MA, this artefact cannot easily be removed by using post-processing techniques such as template subtraction or filtering, commonly used for GA and pulse artefact correction. The effects of the MA are generally therefore ameliorated by removing segments of EEG data that contain large motion artefacts and assuming that neural activity dominates in the remaining data (Bonmassar et al., 2002; Allen et al., 1998). However, in recent years it has been shown that MA can mimic brain activity and cause spurious correlations with fMRI data, which are unrelated to the task of interest. These can cause misinterpretation of data due to spurious, artefactual relationships between EEG MA and BOLD signal if not carefully classified (Fellner et al., 2016; Jansen et al., 2012). The MA is therefore extremely problematic and is the focus of this work.

As a result of this improved understanding of the detrimental effect of MA on EEG-fMRI data quality and relationships between these measures, a number of methods have been developed for removing instances of MA from EEG data. These generally involve recording motion-related reference signals, which are then used to correct the artefact in post-processing. In one approach, a set of wire-loops is used to record motion signals which are then adaptively filtered to match the MA present on each EEG channel before subtraction of the estimated artefact voltages (Masterton et al., 2007; Jorge et al., 2015; van der Meer et al., 2016). An alternative approach involves monitoring the position of a marker attached to the subject's head using a Moiré-phase tracking (MPT) system (LeVan et al., 2013; Maziero et al., 2016). The resulting measurements are then converted into velocities and squared velocities that are combined into a general linear model and regressed out of the EEG time-course. A third type of approach uses an electrically conducting reference layer, which is electrically isolated from the scalp, to record the induced artefacts directly (Chowdhury et al., 2014; Hermans et al., 2016; Steyrl et al., 2017).

Whilst all of these methods have shown promise for artefact correction, none have been shown capable of producing perfect correction of the MA. Furthermore, there has been little characterisation of the efficacy of correction of the MA due to different types of head movement. It is important to understand the precise mechanisms underlying the generation of the MA and whether these are consistent across different types of head movement (e.g. for both nodding and shaking of the head). This knowledge will help in understanding the upper bounds of performance of current techniques for MA correction and can inform the development of improved MA correction methods. Here, we perform a series of experiments with the aim of improving our understanding of the origin of the MA and the current limitations of the reference signal approaches for MA correction. We investigate whether assumptions about rigid body movement are valid for typical head movements and evaluate the effects of relative movements on the MA induced at the scalp compared with the MA induced in a reference layer system.

## Methods

All recordings from healthy human volunteers were made with the approval of our local ethics committee and with informed consent.

### Equipment

Data were acquired in a Philips Achieva 3T MRI scanner (Best, Netherlands) with a 32-channel receive head coil. Measurements of head and EEG cap motion were made using an MR-compatible optical camera (Kineticor, HI, USA) which was attached to the inside of the MRI scanner's bore. The camera concurrently tracked the position and orientation of three, MPT markers with a sampling rate of approximately 80 frames per second and a precision on the order of 10 μm and 0.01° (Maclaren et al., 2012). The position of the camera was carefully adjusted to capture the positions of all three markers simultaneously.

EEG data were acquired using a 32-channel EEG system (Brain Products GmbH, Gilching, Germany) with an MRplus amplifier, using a sampling rate of 5 kHz and a frequency range of 0.016–250 Hz with a 30 dB roll-off at high frequencies. The EEG caps were always placed in the MRI scanner with electrodes Fp1 and Fp2 at isocentre (Mullinger et al., 2011). Two different EEG cap set-ups were used for data acquisition:

i) Standard 32-channel, MR-compatible EEG caps (EasyCap GmbH, Herrsching, Germany). 31 electrodes followed the extended 10–20 system with a reference electrode positioned between Fz and Cz. The caps also had an additional channel for electrooculography, which was attached under the left eye.
ii) RLAS caps, as described in (Chowdhury et al., 2014). Briefly, they consisted of a set of paired electrodes mounted onto an electrically insulating cap. The electrodes in each pair were electrically isolated from one another, but precisely overlaid. One electrode of a pair was connected to the conductive phantom or subject's scalp, while the other was connected to the conductive reference layer. The ground electrode was located at Pz and connected to both the scalp and reference layer, and a pair of reference electrodes were located at Cz. 9 additional pairs of electrodes were sited at locations Fp1, Fp2, O1, Oz, O2, Fc5, Fc6, Cp5 and Cp6. A tight-fitting, conductive reference layer was made from hydrogel (Katecho, Inc., IA, USA) and fixed on-top of the insulating layer of the cap, covering a similar area to the insulating layer, including under the chin. The electrode pairs were connected to the EEG amplifier using star-quad cables (Van-Damme Cable) which were bundled together and fed through a hole in the reference layer at the pole, producing a lead arrangement similar to that used in standard EEG caps.

The solid, conductive, head-shaped phantom used in some of these experiments was formed from kappa carrageenan (4%) in deionised water, doped with NaCl (0.5%) to produce a similar conductivity to brain tissue.

### Measurements of motion utilising a MPT system

A number of experiments were performed to assess the relative motion of the head and EEG caps as follows:

i) To obtain a baseline measure of the accuracy of the MPT system in tracking relative motion of different elements of the EEG cap and head, three MPT markers were affixed using pressure-sensitive scotch tape (3M, MN, USA) to a rigid plastic sphere of 18 cm diameter.
ii) Commercial, standard, EEG caps were fitted to three healthy volunteers. The three MPT markers were fixed to the subject's nasion, the scalp just above the eyebrows and to the material of the EEG cap equidistant between electrodes Fp1 and Fp2 (i.e. at Fpz, although there was no electrode at this position).
iii) RLAS caps were fitted to the conductive head-shaped phantom and a healthy human volunteer. A section of the conducting reference layer between Fp1 and Fp2 was cut away and an MPT marker was affixed to the insulating layer at location Fpz (again there was no electrode at this location). MPT markers were also placed centrally on the forehead, and on the conductive reference layer at the location of electrode Fp1.

The placement of the MPT markers can also be seen in Figure S1. All subject data were acquired with the volunteer's head inside the 32-channel receiver head RF coil whilst wearing the scanner's headphones, to mimic the conditions that obtain during standard EEG-fMRI experiments. Additional padding was used to ensure a snug fit of the subject within the head coil. The top half of the RF coil was removed to ensure easy line-of-sight to the optical camera for recording the marker positions. Motion of the phantom was recorded whilst small, manual rotations were made about the z- or x-axes of the scanner to mimic “shake” and “nod” movements typical of a subject. These rotations were made by an experimenter in the MRI scanner bore physically moving the phantom with their hands whilst receiving verbal feedback from the experimenter in the console room, who continually monitored MPT marker movement. Subjects were given verbal cues to make a sequence of particular head motions consisting of small, repeated head nods or head shakes (oscillating backwards and forwards or side-to-side). For both phantom and subject data, the periods during which a particular type of movement was made, were approximately 1 min in duration and were interleaved with periods of rest (during which subjects were ask to lie as still as possible).

Processing of motion data was carried out using MATLAB (R2016a, MathWorks, USA). Where tracking of the MPT markers was lost due to a marker moving out of the camera frame, data were excluded. MPT marker position data were segmented according to the type of motion.

In order to assess the motion of the head relative to the EEG cap, the Euclidean distances between pairs of markers were calculated for each time point. The Euclidean distance of each marker from the camera was also calculated as a measure of the absolute magnitude of the movement. The time-courses of Euclidean distance variation were high-pass filtered (“brick-wall” spectral filter, high-pass cut-off frequency of 0.2 Hz) to remove baseline off-sets. Root-mean-squared (RMS) Euclidean distances were calculated and averaged over subjects, where applicable, to allow quantitative assessment of the relative motion of the markers in the different cases outlined.

### EEG recordings of motion artefact

Using standard 32-channel MR compatible EEG caps, EEG data were collected for three healthy human subjects concurrently with the MPT-based measurements of position. EEG data were also collected using the RLAS caps on two conductive phantoms and two subjects. One of the phantom data sets and one of the subject data sets were acquired simultaneously with the MPT measurements outlined above.

#### Analysis

EEG data were analysed using BrainVision Analyzer (v2.1, Brain Products GmbH, Gilching, Germany) and MATLAB. Data were band-pass filtered 0.02–10 Hz (frequency range over which the measured MAs dominated) using 8^th^-order, zero-phase Butterworth filters. For data collected using the RLAS system (Chowdhury et al., 2014), reference-layer EEG channels were re-referenced to the electrode paired with the scalp reference electrode that was used as the reference for all channels during the recording. Data for each channel were then baseline corrected by subtraction of the mean signal across time. All EEG data were then segmented into periods capturing rest and the two types of head movements (nod and shake).

MAs were assessed for the scalp electrodes for both types of EEG cap. For the RLAS caps (Chowdhury et al., 2014) MAs were also assessed for the reference layer electrodes and the difference between the reference layer and scalp electrodes (RLAS correction). For each of the MA measures, the RMS values were calculated across time for each electrode and plots of the spatial topography of these values made. Correlation coefficients between scalp and reference layer signals were calculated for each electrode pair, along with the attenuation achieved by using RLAS correction. Spatial maps of correlation and attenuation were also made to identify spatial variability in the efficacy of RLAS correction.

## Results

### Measurements of motion utilising a MPT system

#### Achievable precision to characterise rigid body movement

The relative movements of the MPT markers fixed to the surface of the rigid spherical phantom were first assessed. RMS measures of the high-pass filtered time-courses of each marker's Euclidean distance from the camera and from the other markers are reported in Table 1. The largest change in Euclidean distance to the camera for a single marker was for the simulated head-shake, which showed a maximum movement of 0.422 mm (Table 1A). For the relative movement between markers, the largest RMS value occurred for the shake movement and was measured to be 0.017 mm (Table 1B). This value is in agreement with previous estimates of the precision of the MPT system (Maclaren et al., 2012). Therefore, for our experiments, we chose to consider any changes in relative position between markers greater than 0.017 mm to be indicative of non-rigid body movement.

**Table 1:** Root-Mean-Square (RMS) measures of motion data in mm for markers attached to the rigid sphere. A: shows the RMS of high-pass filtered motion data of individual markers relative to the camera for motions “Rest”, “Nod” and “Shake”. B: shows the RMS of the change of position between pairs of markers for the three types of movement.

**A**			
	**Individual Markers (mm)**		
**Motion**	*Mk1*	*Mk2*	*Mk3*
**Rest**	0.008	0.007	0.009
**Nod**	0.159	0.035	0.199
**Shake**	0.422	0.082	0.406

**B**			
	**Between Markers (mm)**		
**Motion**	*Mk1–Mk2*	*Mk1-Mk3*	*Mk2-Mk3*
**Rest**	0.001	0.002	0.002
**Nod**	0.006	0.012	0.012
**Shake**	0.010	0.010	0.017

#### Motion of head and standard EEG caps

**Fig. 1:**
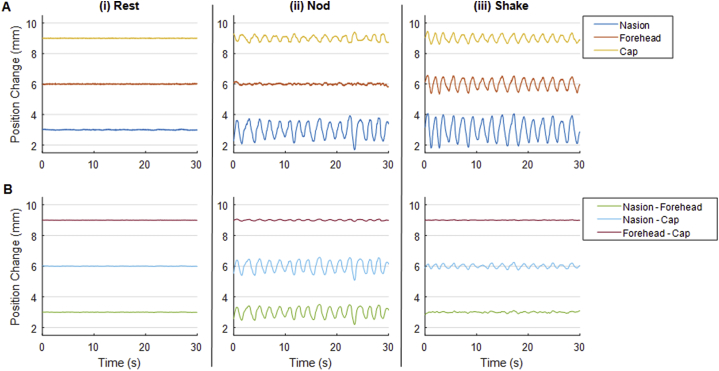
Time-courses of MPT data taken from a representative subject wearing a standard 32 channel EEG cap. MPT markers were fixed to the nasion, centre of the forehead and the cap at the Fpz position. Panel A shows the change in position of individual markers relative to the camera frame and panel B shows the change in position between pairs of markers. Types of movement are separated column-wise showing: (i) a rest period, (ii) a continuous head-nod motion period and (iii) a period of head-shake. Note, a 3 mm offset between time-course baselines is employed to aid visualisation in all plots.

Time-courses of relative motions of the standard EEG cap, the scalp and nasion of a representative subject are shown in Fig. 1. Table 2 shows RMS measures of the relative motion data averaged over all subjects. The data collected from the rest period (Fig. 1Ai and Table 2A) show little appreciable motion of individual markers relative to the camera, as expected. However, the RMS values acquired during this rest period are 2–3 times larger than the corresponding measures on the rigid phantom (Table 1A), likely due to head movement related to the cardiac (Yan et al., 2010) and respiratory cycles. It is clear from Fig. 1Aiii and Table 2, that the shake motion elicits the largest physical movement of all three markers relative to the camera, with the marker attached to the nasion (dark blue) moving the most. However, considering the movement of markers relative to one another, the largest deviations are seen for the nod movement (Fig. 1Bii and Table 2), with the movement of the nasion marker compared with the markers on the forehead and cap being the largest. Whilst no appreciable movement of the markers on the forehead and cap relative to one another was observed for a head shake, small relative movement of these markers was seen for a head nod. Similarly, small relative motion was observed for a head shake when considering the movement of the nasion relative to that of the other monitored positions.

**Table 2:** Root-Mean-Square (RMS) measures of motion data in mm for subjects wearing the standard MR compatible 32-channel EEG cap. Mean (standard deviation) of RMS measures calculated across the three subjects. A: Shows the RMS high-pass filtered motion data of individual markers, attached to the nasion, centre of the forehead and on the EEG cap at location Fpz, relative to the camera for “Rest”, “Nod” and “Shake” movements. B: shows the RMS of the change of position between pairs of markers for the three types of movement. Note that coloured heading highlights the measures that can be directly compared with those acquired with the RLAS EEG cap (Table 3).

**A**				
**Motion**	**Nasion**	**Forehead**		**Cap**
**Rest**	0.03 (0.02)	0.022 (0.008)		0.025 (0.002)
**Nod**	0.2 (0.2)	0.04 (0.01)		0.13 (0.02)
**Shake**	0.4 (0.2)	0.23 (0.06)		0.17 (0.05)

**B**				
**Motion**	**Nasion – Forehead**		**Nasion – Cap**	**Forehead – Cap**
**Rest**	0.02 (0.04)		0.03 (0.03)	0.01 (0.02)
**Nod**	0.2 (0.1)		0.2 (0.1)	0.04 (0.02)
**Shake**	0.027 (0.008)		0.06 (0.03)	0.015 (0.004)

With markers only attached to the skin surface it is difficult to draw firm conclusions about the relative motion of the skin and skull for different types of head movement. We therefore performed a further investigation to monitor the movement of MPT markers fixed on the skin of the forehead and the nasion, relative to a third marker attached to a bite-bar mounting, constructed from PVC and fitted to each subject using dental impression compound (Kerr Corporation, CA, USA). The results of this investigation showed that the greatest relative motion was between the forehead and bite-bar, with a smaller relative motion between the nasion and bite-bar (see Supplementary Results, Figure S2 and Table S1).

#### Motion of RLAS system

**Fig. 2:**
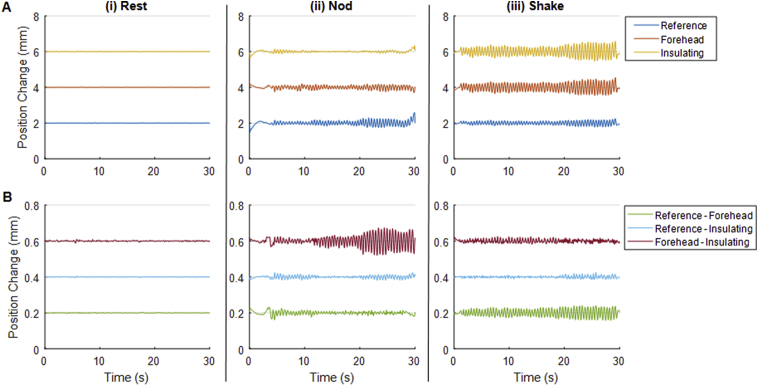
Time-courses of MPT data recorded from a gel phantom onto which an RLAS cap was attached. MPT markers were affixed to the centre of the forehead of the phantom, the insulating layer of the cap at the Fpz location and the reference layer of the cap at the Fp1 location. Panel A shows the change in position of individual markers relative to the camera frame and panel B shows the change in position between pairs of markers. Types of movement are separated column-wise showing: (i) recordings during the rest period, (ii) recordings during a continuous head-nod like motion and (iii) a period of head-shake. Note, a 2/0.2 mm offset between time-course baselines is employed to aid visualisation in panels A and B, respectively.

**Fig. 3:**
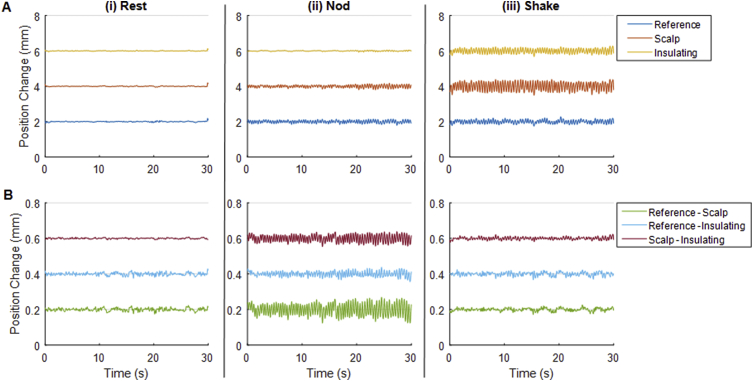
Time-courses of MPT data recorded from a healthy human subject wearing an RLAS cap. MPT markers were affixed to the centre of the forehead of the subject, the insulating layer of the cap at the Fpz location and the reference layer of the cap at the Fp1 location. Panel A shows the change in position of individual markers relative to the camera frame and panel B shows the change in position between pairs of markers. Types of movement are separated column-wise showing: (i) recordings during the rest period, (ii) recordings during a continuous head-nod motion and (iii) a period of head-shake. Note, a 2/0.2 mm offset between time-course baselines is employed to aid visualisation in panels A and B, respectively.

Results of monitoring the motion of the RLAS system on the phantom and subject are shown in Fig. 2, Fig. 3, respectively, and Table 3. As expected, very little motion of individual markers was detected in the rest period (Table 3A) and no significant change in relative positions between markers was observed (Table 3B, row 1) for both the phantom and the subject. The RMS measures in Table 3A indicate that the size of the “nod” and “shake” motions are comparable between the phantom and subject, and also comparable to those found with the standard cap (Table 2, Table 3A, forehead measures). For all movements on the phantom and subject, little relative movement of the insulating and reference layers was observed indicating that these layers are well coupled. The “shake” motion generated no clear variation in the relative position between pairs of MPT markers for the phantom or subject (Table 3B, row 3). The largest changes in relative position between markers were observed for both phantom and subject recordings made with nodding motion. For the phantom, a notable variation in the Euclidean distances between the markers attached to the forehead and insulating layer of the RLAS cap was observed. For the subject, changes in the position of the marker attached to the subject's forehead relative to the markers attached to the reference and insulating layers of the RLAS cap were significantly different from zero. For all movement types the relative movement of the forehead and insulating layer were comparable for the RLAS system measures on the phantom and subject, and much smaller than for similar measures on the 32-channel cap (compare Table 2, Table 3B, red headings).

**Table 3:** Root-Mean-Square (RMS) measures of motion data in mm for the gel phantom and subject wearing an RLAS cap. Mean of RMS measures calculated across the three recordings. A: Shows the RMS of high-pass filtered motion data of individual markers attached to the centre of the forehead (scalp), the insulating layer at the position of Fpz and the reference layer at the position of Fp1 relative to the camera for “Rest”, “Nod” and “Shake” movements. B: shows the RMS of the change of position between pairs of markers for the three types of movement. Note that coloured heading highlights the measures that can be directly compared with those acquired with the standard EEG cap (Table 2).

**A**						
	**Phantom**			**Subject**		
**Motion**	*Ref*	*For*	*Ins*	*Ref*	*For*	*Ins*
**Rest**	0.005	0.005	0.007	0.032	0.031	0.022
**Nod**	0.089	0.058	0.053	0.072	0.061	0.019
**Shake**	0.069	0.161	0.173	0.084	0.191	0.108

**B**						
	**Phantom**			**Subject**		
**Motion**	*Ref-For*	*Ref-Ins*	*For-Ins*	*Ref-For*	*Ref-Ins*	*For-Ins*
**Rest**	0.001	0.001	0.002	0.008	0.008	0.003
**Nod**	0.009	0.006	0.019	0.026	0.012	0.017
**Shake**	0.016	0.005	0.007	0.013	0.010	0.007

### EEG recordings of motion artefact

**Fig. 4:**
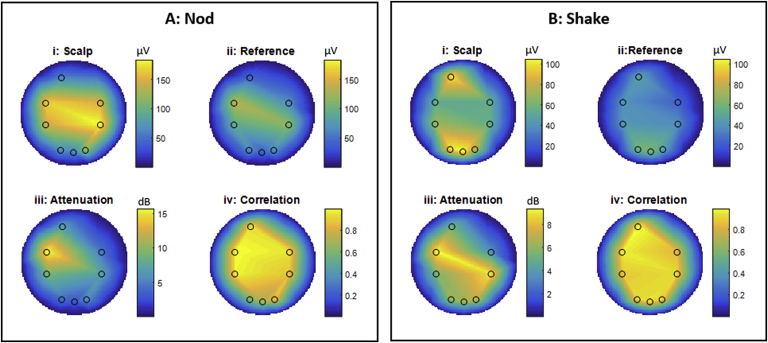
Flat-maps showing the results from EEG experiments on gel phantoms onto which the RLAS cap had been placed. The values shown are calculated as an average across all three recordings acquired from two separate sessions. Box A shows the results for a nod motion, where box B displays the results from a shake motion. i and ii show the RMS values of the EEG data for the channels connected directly to the phantom (scalp) and the isolated reference layer, respectively. iii show the attenuation of the scalp artefacts produced by directly subtracting the reference layer recording and iv show the correlation coefficients between timecourses used to generate i and ii. Note the difference in colour-bar scales for head nod and shake movements.

**Fig. 5:**
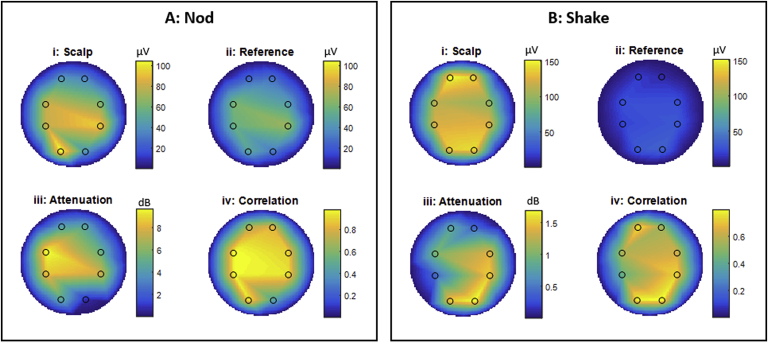
Flat-maps showing the results from EEG experiments on human subjects wearing the RLAS cap. The values shown are calculated as an average across three recordings made in two separate sessions. Box A shows the results for a nod motion, where box B displays the results from a shake motion. i and ii show the RMS values of the EEG data for the channels connected directly to the subject's scalp and the isolated reference layer, respectively. iii shows the attenuations of the scalp artefacts produced by directly subtracting the reference layer recording and iv show the correlation coefficients between timecourses used to generate i and ii. Note the difference in colour-bar scales for head nod and shake movements.

Recordings made from channel Fp2 for the phantom and channel Oz for the subject wearing the RLAS cap were excluded from the analysis due to faulty electrode pairs. Fig. 4, Fig. 5 show the spatial variation of the RMS amplitude of the induced voltages in the phantom and subject recordings, respectively. These spatial maps show a general right-left artefact topography (in which the RMS voltages are largest in the most lateral [right/left] electrodes) for a head nod and an anterior-posterior topography (in which the RMS voltages are largest in the most anterior and posterior electrodes) for a head shake on the scalp, with a similar, but significantly smaller amplitude pattern, on the reference layer for both the phantom and subject (p < 0.05, paired *t*-test across scalp-reference electrode pairs, for all movements on the phantom and subject). The RMS values of the voltages on the two layers are reported in Table 4, Table 5 for the phantom and subject. These patterns are similar to those observed when using the standard EEG caps, where a greater number of sampling locations provides a clearer depiction of the artefact morphology (see Figure S3). For both the phantom and human data sets, the EEG artefacts due head motion are much greater in amplitude than most neuronal signals of interest.

**Table 4:** The average values and standard deviations, in brackets, of the results from the EEG experiments across the three recordings of the gel phantom wearing the RLAS cap. A shows the results for the nod motion and B shows the results for the shake motion. Labels i, ii, iii and iv directly link to the results depicted in Fig. 4. Average and standard deviations at the bottom of each table are calculated across electrodes.

**A: Phantom Nod**					
	**RMS (μV)**				
**Electrode**	*Scalp (i)*	*Reference (ii)*	*RLAS*	*Atten-dB (iii)*	*Corr (iv)*
**Fp1**	59 (18)	42 (19)	28 (7)	6.5 (0.5)	0.94 (0.06)
**Fc5**	166 (79)	133 (92)	35 (31)	15.8 (12.7)	1.00 (0.00)
**Cp5**	144 (68)	92 (69)	58 (46)	9.0 (7.9)	0.98 (0.02)
**O1**	70 (41)	65 (53)	41 (17)	4.2 (6.8)	0.61 (0.32)
**Oz**	59 (30)	41 (25)	40 (19)	2.9 (2.2)	0.70 (0.19)
**O2**	120 (82)	69 (63)	54 (29)	5.7 (4.4)	0.88 (0.17)
**Cp6**	183 (123)	116 (127)	74 (34)	7.0 (7.4)	0.88 (0.13)
**Fc6**	149 (74)	65 (65)	86 (36)	4.3 (4.2)	0.95 (0.06)
**Average**	119 (50)	78 (33)	52 (20)	6.9 (4.1)	0.87 (0.14)

**B: Phantom Shake**					
	**RMS (μV)**				
**Electrode**	*Scalp (i)*	*Reference (ii)*	*RLAS*	*Atten-dB (iii)*	*Corr (iv)*
**Fp1**	101 (41)	46 (20)	56 (21)	5.1 (0.3)	0.99 (0.01)
**Fc5**	53 (27)	39 (19)	17 (7)	9.4 (1.6)	0.96 (0.04)
**Cp5**	69 (53)	42 (35)	32 (14)	5.6 (2.8)	0.88 (0.12)
**O1**	100 (34)	60 (21)	48 (16)	6.3 (1.8)	0.93 (0.07)
**Oz**	109 (39)	61 (28)	50 (15)	6.3 (2.2)	0.93 (0.07)
**O2**	79 (20)	47 (30)	37 (16)	7.0 (6.0)	0.90 (0.09)
**Cp6**	55 (24)	37 (12)	24 (18)	8.9 (5.4)	0.92 (0.07)
**Fc6**	54 (25)	24 (10)	36 (13)	3.3 (0.9)	0.83 (0.15)
**Average**	77 (23)	45 (12)	37 (13)	6.2 (2.0)	0.92 (0.05)

**Table 5:** The average values and standard deviations, shown in brackets, of the results from the EEG experiments three recordings with a subject wearing the RLAS cap. A shows the results for the nod motion and B shows the results for the shake motion. Labels i, ii, iii and iv directly link to the results depicted in Fig. 5. Average and standard deviations at the bottom of each table are calculated across electrodes.

**A: Subject Nod**					
	**RMS (μV)**				
**Electrode**	*Scalp (i)*	*Reference (ii)*	*RLAS*	*Atten-dB (iii)*	*Corr (iv)*
**Fp1**	41 (13)	24 (12)	25 (4)	4.1 (3.0)	0.72 (0.31)
**Fc5**	81 (8)	61 (22)	27 (9)	9.7 (3.2)	0.98 (0.01)
**Cp5**	80 (15)	56 (21)	30 (5)	8.5 (2.9)	0.97 (0.01)
**O1**	106 (14)	49 (5)	62 (14)	4.7 (1.1)	0.94 (0.02)
**O2**	41 (7)	31 (12)	40 (16)	0.7 (2.3)	0.45 (0.31)
**Cp6**	94 (83)	69 (42)	39 (32)	7.6 (2.1)	0.93 (0.07)
**Fc6**	64 (43)	45 (11)	35 (20)	5.1 (0.9)	0.89 (0.09)
**Fp2**	35 (7)	28 (15)	21 (2)	4.4 (1.3)	0.81 (0.08)
**Average**	68 (27)	45 (17)	35 (13)	5.6 (2.9)	0.83 (0.18)

**B: Subject Shake**					
	**RMS (μV)**				
**Electrode**	*Scalp (i)*	*Reference (ii)*	*RLAS*	*Atten-dB (iii)*	*Corr (iv)*
**Fp1**	152 (35)	17 (4)	139 (33)	0.8 (0.4)	0.75 (0.24)
**Fc5**	112 (43)	23 (5)	100 (38)	1.0 (0.6)	0.57 (0.24)
**Cp5**	122 (42)	20 (5)	116 (37)	0.4 (0.7)	0.43 (0.49)
**O1**	137 (24)	30 (11)	114 (10)	1.5 (0.7)	0.79 (0.19)
**O2**	143 (24)	34 (15)	116 (6)	1.7 (1.0)	0.80 (0.21)
**Cp6**	126 (28)	28 (4)	107 (33)	1.5 (0.8)	0.73 (0.19)
**Fc6**	98 (31)	24 (4)	85 (34)	1.4 (0.9)	0.63 (0.22)
**Fp2**	139 (28)	11 (2)	134 (30)	0.4 (0.3)	0.54 (0.17)
**Average**	129 (18)	23 (8)	114 (17)	1.1 (0.5)	0.66 (0.13)

The discrepancy in the magnitude of the artefacts induced in the two layers is illustrated by the relatively large residual artefacts (Table 4, Table 5, RLAS column) and low attenuations (Fig. 4, Fig. 5(iii) and Table 4, Table 5(iii)). The average RLAS attenuation performance over the electrodes is however similar for the nod (6.9 dB) and shake (6.2 dB) movements on the phantom. On the subject the attenuation is slightly reduced for the nod movement (5.6 dB) and considerably less for a shake movement (1.1 dB) compared with the phantom. Interestingly, despite these low attenuation values the correlation of the artefact waveforms from the scalp and insulating layers are high (Fig. 4, Fig. 5 (iv) and Table 4, Table 5(iv)) for the head nod and shake movements for the phantom (0.87 and 0.92, respectively). In the case of the human subject data, good correlation of the artefacts measured from the scalp and reference layer is also seen for the nodding movement (0.83) however, the correlation between MA waveforms is considerably reduced for the shake movement (0.66). These results show that in general there is a good spatial and temporal correlation between the MA induced on the reference layer and scalp, but the amplitudes of the artefacts always differ considerably.

Table S1 shows the RMS over time of the potential difference between the recordings on the reference pair of electrodes (one connected to the scalp and the other connected to the reference layer). The difference in induced artefact between layers is small in the phantom (<30 μV), but significantly larger (∼115 μV) for the head shake motion in the data from the subject.

## Discussion

Here, we used an MPT system to assess the movements of the skull, scalp and EEG cap which underlie the MAs that affect EEG data measured during simultaneous fMRI. We focus on whether there is evidence for non-rigid body motion of the EEG-cap/head system, as this provides useful information in considering the efficacy of different approaches to MA correction. Reference-signal-based approaches to motion artefact correction (LeVan et al., 2013; Masterton et al., 2007; Jorge et al., 2015; van der Meer et al., 2016; Maziero et al., 2016; Chowdhury et al., 2014; Hermans et al., 2016; Steyrl et al., 2017) are likely to deliver better artefact reduction and to be simpler to implement if all elements of the head/EEG cap system move together as a rigid body. We relate the relative movements of the system components to the induced MA that we observe in EEG recordings from a conventional EEG cap and an RLAS set-up.

Here, we show that there is considerable movement of the skin relative to the skull when a head movement takes place, with this non-rigid body movement being larger for a head nod than shake. We find the movement of the EEG cap relative to the phantom or skin on the forehead is comparatively small. Given the lack of relative movement of the skin, EEG cap and reference layer we find the surprising result of large differences in the amplitude of the MA induced voltages on EEG electrodes attached to the scalp/phantom compared with the reference layer. We also observe, surprisingly, that on a human these differences are considerably larger for a head shake movement than head nod. Below we discuss the likely origin of these findings, interpretation and relevance for the future development of MA correction methods.

### Motion monitoring using the MPT system

In agreement with previous investigations of head movement for motion correction of MRI data (Singh et al., 2015) we found considerable relative movement between the markers at the nasion and the forehead locations (Table 2), which probably indicates that the scalp and skull do not move together as a single rigid body when the head moves with the subject lying inside an MRI scanner. However, with just two markers that are both attached to the skin surface it is difficult to draw firm conclusions about the relative motion of the skin and skull for different types of head movement. Our additional experiments using a bitebar to monitor skull movement showed that the greatest relative motion was between the forehead and bite-bar, with a smaller relative motion between the nasion and bite-bar (Figure S2 and Table S1), in agreement with previous studies (Callaghan et al., 2015). These data strongly suggest that the relative motion of the nasion and forehead, and of the nasion and EEG cap, reported in Table 2 are due to slipping of the scalp over the skull, which we observed to be larger for a head nod than a head shake movement. This has important implications for the efficacy of MA correction if MPT markers are to be used in the correction (LeVan et al., 2013; Maziero et al., 2016), as the marker placement is likely to have significant effects on correction performance.

We also observed some movement of the EEG cap relative to the subject's forehead when a standard EEG cap was used (Fig. 1 and Table 2) and the subject performed small head nods. A similar effect was observed for the RLAS system, although the relative motion was smaller (Table 3). The motion of the cap relative to the forehead for both EEG caps was less than the threshold value of 0.017 mm for head shaking, although relative motion was again smaller for the RLAS system. These results likely reflect the fact that the standard EEG caps have a poorer fit to the head as, in contrast to the RLAS caps, they were not custom-made for each subject. These results suggest that a close-fitting cap may be advantageous for the correction of the MA using reference-signal-based methods.

Our data also revealed that there was some motion of the reference layer of the RLAS system relative to the skin when a head nod was performed, despite a tight coupling of the insulating and reference layers of this cap (Table 3). This is likely to be the combined effect of a small slipping of the EEG cap relative to the skin and a further slipping of the two layers relative to one another. However, these relative movements were still only 0.009 mm larger than our threshold of accuracy and approximately a factor of two smaller than the movements of the standard EEG cap relative to the forehead.

The contribution of the skin-skull movement obviously cannot explain the non-rigid body movement observed for the nodding movement of the phantom and cap (Table 3). To test whether this motion was caused by deformation of the phantom, unrelated to the presence of the EEG cap, an additional experiment was performed in which the three MPT markers were fixed to carriers which were pushed into the gel phantom and placed on the front of the phantom. The results (Table S2) indicated that, changes in the distances between markers were less than the 0.017 mm threshold for all types of movement. This suggests that there was no deformation of the gel phantom. Instead, in the case of both the phantom and subject, the relative movement between the marker on the forehead or that attached directly to the phantom and the markers on the cap is likely to be due to a small relative slipping of the layers, which is smaller for the RLAS system than the standard EEG cap.

Whilst cable path deformation was not monitored in our experiments it is likely, that as previously discussed (LeVan et al., 2013), there is also deformation of the wires connecting the EEG electrodes and amplifier when the head or phantom moves. The additional artefacts that this deformation generates will have varying temporal profiles and are likely to add additional challenges in fully correcting the MA when a standard EEG cap is used. However, in the RLAS system where star-quad cable is used to connect pairs of electrodes to the EEG amplifier, the effect of wire movement should be largely negated (Chowdhury et al., 2014) and therefore further consideration of the motion of the wires was not made in this study.

The degree of non-rigid body movement is likely to be proportional to the overall amplitude of the movements that take place. In this study we aimed to emulate the magnitude and rate of movements typically observed in EEG-fMRI studies, but to increase the frequency of these movements to aid our study and understanding of the MA. Panel A of Fig. 1, Fig. 2, Fig. 3 show that this was achieved. Subject data collected with the standard cap (Fig. 1) and RLAS cap (Fig. 3) showed a maximum peak-to-peak motion (relative to the initial position of the head) for both the head nod and shake of ∼2mm/<1 mm for the standard/RLAS cap datasets, respectively. The phantom movement, wearing the RLAS cap, showed a peak-to-peak movement amplitude of <1 mm for a nod and ∼1 mm for the shake (Fig. 2). Therefore the magnitudes of the movements made in this study are well within the maximum movement commonly observed in standard EEG-fMRI experiments. Given that fMRI data analysis, would typically only exclude data if the realignment parameters revealed motion greater than the voxel size of the fMRI acquisition, it is likely all our data would be included, based on the fMRI acquisition. It would be helpful to be able to use realignment parameters from fMRI data to guide when the non-rigid body movement of the head has occurred and the required exclusion of EEG data from typical EEG-fMRI experiments. However, small, rapid head movements cannot be detected using these parameters due to insufficient temporal resolution, as previously discussed (Fellner et al., 2016; Jansen et al., 2012), and therefore we cannot recommend that fMRI realignment parameters can be used to guide the quality of the EEG data or whether MA and non-rigid body motion is a significant problem in a given dataset.

#### Limitations of movement monitoring

While care has been taken to ensure accurate tracking of the motion of the head and EEG caps, there are inherent limitations associated with our approach. In particular, the small field-of-view of the camera and requirement for line-of-sight to all markers limited the locations at which markers could be placed. It is therefore possible that some complexities of the motion of the head and EEG system are not characterised in our measures: for example, it was not possible to monitor movement of the EEG cap at the back of the head where it rests on the RF coil.

In addition, we have not attempted to characterise the effects of other types of head movement, such as jaw clenching and making facial expressions, which may also cause small movements of the EEG electrodes and different forms of non-rigid body movement. We did not characterise these movements as they are associated with large muscle artefacts (e.g (Goncharova et al., 2003)) which will only be detected by the scalp layer electrodes and therefore cannot be corrected fully with any sort of reference layer/signal based system. Thus they add significant additional complexity to the artefact problem, which is beyond the scope of this work, but does warrant future investigation.

### MA induced in RLAS system

As expected, the MA induced in the scalp layer had a right-left topography for a head nod and an anterior-posterior topography for a head shake (Fig. 4, Fig. 5 and S3), in agreement with previous reports (Yan et al., 2010). A similar spatial topography was observed for the reference layer, but the amplitudes of the artefacts on reference layer electrodes were significantly smaller, both for the phantom and subject recordings. This discrepancy in the MA on the two layers was noticeably worse for the subject executing a head shaking motion compared with head nodding.

#### Phantom

The phantom data revealed similar levels of attenuation and correlation of the artefacts between the scalp and reference layers for both shaking and nodding movements (Fig. 4, Table 4). This is despite the larger difference in the motion of the layers of the RLAS cap relative to the forehead when a head nod was performed compared with a head shake, which might be expected to produce a larger discrepancy in the MA on the two layers for a head nod. Differences in the MA induced in the electrode pairs are therefore unlikely to be explained by movement of the cap relative to the phantom.

It is important to note that the attenuation of the MA found using RLAS here (Table 4) is far lower than we previously reported for data acquired on a phantom (mean attenuation 30 dB (Chowdhury et al., 2014),). This reduction in the attenuation achieved is due to a change in the RLAS cap configuration. Previously the star-quad cables were “routed under the gel layer before exiting from the right or left side of the phantom/subject, coming together under the chin”, while here, the RLAS cap was modified for convenience of use, and the star-quad cables followed the routing used in a standard EEG cap with the wires leaving at the crown of the head and being bundled from this point. The MA recorded on an electrode includes a contribution from the EEG leads (Yan et al., 2010) whose magnitude depends on the area of the loop that is effectively formed by the lead attached to the electrode of interest and the lead attached to the reference electrode. When RLAS is applied, this contribution to the artefact is eliminated because the use of the star-quad cable means that the leads attached to a pair of scalp and reference layer electrodes follow exactly the same path producing almost identical current loops. In the previous RLAS cap set up (Chowdhury et al., 2014) the loops formed by the scalp electrode and scalp reference electrode leads were much larger than in the new set-up because of the difference in wire routing. As a consequence, the MA peak-to-peak voltages on scalp electrodes were >10 mV in magnitude in the previous configuration, compared with a maximum of ∼1 mV observed here. Since in both cases, subtraction of the reference layer signals almost completely eliminates the lead contribution to the MA, the attenuation in dB was much higher for the previous set-up. Here, before RLAS correction the RMS MA was 119 μV (Table 4) compared with around 1500 μV in the previous study (estimated from (Chowdhury et al., 2014)), so that using the measured attenuations of ∼7 dB and ∼29 dB we find a residual RMS MA after RLAS of around 50 μV in both cases. The mismatch in MA amplitude on reference layer and scalp is thus likely to be similar in the two different RLAS set-ups and merits further investigation.

Assuming that the artefacts in the star-quad cable are perfectly cancelled, the only contribution from the wires would be from the small section of wire required between the two electrodes before the same wire path can be followed. Care was taken to make the areas of wire loops at the junction between the star-quad cable and the electrode pair as small as possible (<0.8 cm^2^). Assuming these wire loops are rotating at 1°/sec then the magnitude of the induced voltage would be < 2.06 μV in amplitude at 3T, and so unlikely to explain the measurements observed.

Although the conductivity of the reference layer and phantom were not perfectly matched (as they were made of different materials) we do not believe that this imperfect matching causes the difference in the MA induced in the two layers. Bench testing of the conductivity showed that the conductivity of the hydrogel used to make the reference layer was about 5 times less than that of the kappa carrageenan gel, used to make the phantom. Since the conductivity of the latter is similar to that of physiological saline the reference layer is a good enough conductor to yield similar induced electric field to those generated in the head or phantom when exposed to time-varying fields due to head motion or applied gradients in MRI (Yan et al., 2009, 2010). In separate data sets collected on the phantom (in one recording) and on the human subjects (in 3 recordings) with EPI scanning performed and no movement, the resultant GA after RLAS was attenuated on average over electrodes by 10.5 ± 5.1 dB and 8.8 ± 5.1 dB for the phantom and human data, respectively. As these attenuations are greater than those achieved for the MA (Table 4, Table 5) we do not think the conductivity differences are the cause of the difference in the MA induced on the scalp and reference layers.

An alternative plausible explanation is that the voltages induced in the reference layer by rotation do not closely match those generated at the surface of the phantom. This may be a consequence of the limited extent of the reference layer, which primarily covers one hemisphere of the phantom and has a hole at the pole to allow the wires to leave the cap. Since the induced current in the reference layer must flow azimuthally at the edges of the reference layer, but this condition does not apply to the volume conductor, the variation of potential difference on the reference layer is likely to be perturbed. In these circumstances there is a good temporal correlation of the artefact waveforms induced on the reference layer, with those found at the surface of the phantom or on the scalp surface, but the amplitudes of the MA at individual reference layer and scalp electrode pairs are dissimilar. By fitting the artefact recorded at the reference layer to that measured on the scalp/phantom an appropriate scaling factor can be identified and the fitted version of the artefact can be subtracted to produce better artefact attenuation (Steyrl et al., 2017).

#### Human

The data acquired on the subjects shows a similar pattern for a head nod to the phantom data, and the sources of discrepancy between the layers are likely to be similar to those on the phantom. The slightly smaller RLAS attenuation and lesser correlation of the voltages recorded on the scalp and reference layers in the subject data than the phantom data for the head nod is likely to be explained by the voltages due to brain activity which are recorded on the scalp electrodes in the subject data.

However, these subject data introduce a further paradox as larger relative movements of the forehead and EEG cap are observed for the head nod, but the larger discrepancy in the magnitude of artefacts on the scalp and reference layer is found for the head shake. Given the similarity in the relative movements of the “forehead” and EEG layers for head shake on the phantom and subject (Table 3B), the larger difference in the amplitude of the artefact on the scalp and reference layers in the subject data (Table 5B compared with 4B), and the lower correlation of the two measurements, for head shake in the subject data cannot be explained by relative motion of the forehead and the cap. However, in the subject data, unlike the phantom data, there is a large relative movement of the skin with respect to the skull (Table S1, forehead-bitebar), and consequently the brain effectively rotates with respect to the skin. The brain, which acts as a volume conductor is therefore rotating in the magnetic field by a greater amount than the skin, electrodes and reference layer. In this case a potential difference between the reference electrode located near the axis of rotation, and the scalp electrodes located at a distance from the axis of rotation will be induced. Thus, the head is effectively acting as a homopolar generator in this scenario (Yan et al., 2010). This could lead to a much larger and more complex artefact being produced on the electrode on the scalp layer than can be directly characterised by the paired electrode on the reference layer. As a consequence, the MAs on the scalp and reference layer have a lower correlation and are significantly different in amplitude, meaning that the RLAS attenuation is further reduced. This effect arises because the reference layer movement is coupled with the respective electrodes and therefore there is no homopolar contribution on the reference layer, assuming no slipping of the reference layer relative to the insulating layer, which our MPT data suggests. For further discussion of homopolar generator effect on the MA see the Supplementary Discussion.

A putative alternative explanation for the observed discrepancies in the voltages measured between the scalp and reference layer, is that neuronal signals are induced by the changes in magnetic field. Both a metallic taste (Cavin et al., 2007) and vertigo sensation (Glover et al., 2007) have been reported during head movements in MRI scanners. Furthermore, metallic tastes have been reported to be stronger for a head shake than a head nod (Cavin et al., 2007). It is therefore possible that the discrepancies we observe between the voltages measured on scalp and reference layer during a head shake are in part due to neuronal signatures of these sensations. To our knowledge, the neuronal signatures measured by EEG relating to these sensations are unknown. However, in general the largest brain signals measured with scalp EEG are up to ∼200 μV peak-to-peak (in the alpha band) and typically observed over the occipital cortex. Therefore, we believe it is unlikely that the neuronal signatures of metallic taste or vertigo sensations will have sufficient amplitude, or the required anterior-posterior spatial topography, to explain the patterns of induced voltages by a head shake on the scalp (Fig. 4, Fig. 5Bi), and the discrepancy with those measured on the reference layer (Fig. 5Bii).

### Study limitations and future investigations

This work focused solely on understanding the origin of the MA and the ability to accurately characterise this artefact using a reference layer approach. Changes in head position will also cause changes in the gradient artefact due to the position of the EEG leads, electrodes and head changing relative to the spatially-varying magnetic fields (Yan et al., 2010; Mullinger et al., 2011). It is well known that changes in head position, such that a subject moves to a new position and then remains in that position for a period of time, affect the correction of the GA using standard template correction methods (Moosmann et al., 2009). However, if the timing of the movement is known, then a new template can be formed at each movement occurrence as required (Moosmann et al., 2009) or alternatively GA occurrences can be grouped according to the similarity of artefact profiles e.g. (Freyer et al., 2009). Furthermore, small rapid movements may not cause a noticeable change in GA as the head may effectively move and then return to its original position between successive GA occurrences (e.g. in the case of the head movement related to the cardiac cycle (Yan et al., 2009, 2010)). It is therefore important to consider MAs alone, as done here, before the interaction with the GA artefact is considered. Whilst, the interaction of the GA with the MA is beyond the scope of this paper, the changes in GA should also theoretically be captured by using a reference layer approach, such as RLAS (Chowdhury et al., 2014) and these warrant further investigation in the future. Such work would be needed to ascertain whether the differences in residual MA depending on head movement type carry through to differences in residual GA.

### Implications for correction of motion artefacts

The results presented in this paper suggest that the MA in EEG-fMRI experiments is more complicated than previously thought and not simply dominated by the movement of the leads in the magnetic field. Voltages induced in the volume conductor (head) also provide a large contribution to the overall MA. Even in the case of relatively rigid body motion of the whole system (RLAS cap on the phantom) we found it was not possible to induce perfectly matching voltages in recordings from the scalp and reference layer electrodes, although there was a strong correlation between the waveforms recorded on the two layers. In addition, the relative movement of the head and standard EEG cap which we identified may also contribute to a more complicated artefact formation and will reduce correlation between reference signals (e.g. from wire loops attached to the EEG cap (Masterton et al., 2007)) and the scalp recordings to be corrected.

Reference-layer based correction methods, such as RLAS, in which a direct subtraction of paired recordings is designed to correct for the MA, assume that the reference layer and head move together as a single rigid body, and that artefact voltages induced on the surface of the conducting reference layer are then identical in magnitude to those induced on the surface of the scalp (Chowdhury et al., 2014). However, our data demonstrate that even in the best case scenario of relatively rigid body motion of the system and strong correlation of the artefact voltages on the two layers (Table 4), RLAS correction of the artefacts can still be poor due to the discrepancy in the amplitudes of the voltages on the two layers. The effect of this difference in amplitude can easily be overcome by fitting the reference layer signals to the scalp recordings and this is likely to explain the success of a number of previous studies where fitting approaches based on the use of reference signals acquired either through an RLAS type approach (Hermans et al., 2016; Steyrl et al., 2017), or by using wire loops to capture the motion related artefacts (Masterton et al., 2007; Jorge et al., 2015; van der Meer et al., 2016; Hermans et al., 2016) have been employed. Indeed, with simple, non-adaptive, least-squares fitting of the reference signal to the corresponding scalp signal on the phantom data acquired in this study, the mean residual artefacts could be reduced to 30 (13) μV for nod movements and 24 (8) μV for shake movements, corresponding to a 12 (7) dB and 12 (3) dB attenuation, respectively. The advantage of the RLAS type approach even when fitting is required is that only one reference layer signal needs to be fitted to each scalp channel (e.g. (Hermans et al., 2016; Steyrl et al., 2017)) and therefore over-fitting is conceptually less likely than when voltages induced multiple motion monitors (i.e. wire loops) are used to capture motion information and all of these voltages used in the correction of MA at each electrode location (Masterton et al., 2007; Jorge et al., 2015). However, if it were possible to only have one weighting per motion sensor (i.e. reference layer electrode or wire loop), even using a multi-sensor fit, then over-fitting is unlikely to cause a significant problem due to the number of time points in a typical EEG recording, as previously shown when considering the correction of the pulse artefact (Luo et al., 2014). However, fitting algorithms to correct for all head motion types are generally adaptive, due to the different spatial patterns of the MAs induced by different types of head movement (as shown in Fig. 4, Fig. 5). Therefore over-fitting is still possible, due to the smaller segments of data that are fitted at any point in time, combined with the multiple motion sensor signals often used. Whilst initial studies have indicated adaptive fitting algorithms result in limited brain signal losses (Masterton et al., 2007; Jorge et al., 2015; Maziero et al., 2016; Steyrl et al., 2017), a quantitative investigation on the true effects of such fitting on neuronal data is yet to be published.

The findings of this current study therefore raise the question as to whether gains can be achieved by RLAS type methods compared with the use of simpler reference signals based on wire loops or MPT-based motion parameters. A recent study compared the performance of wire loops to a reference layer system (Hermans et al., 2016). They concluded that the results were comparable, with the reference layer performing marginally better for correcting the MA. However, the reference layer system employed in that study was substantially different in geometry to the RLAS system used here (Chowdhury et al., 2014) and the performance of the correction methods was not compared using the same data, limiting the range of comparisons that were possible.

Our data however, provide evidence for an additional challenge for the correction of MA on the human head. The MA induced by a head nod is likely to be corrected by the fitting methods described above, due to the similar nature of the signal to that measured on the phantom. However, for a head shake an additional discrepancy is introduced which has a considerable effect on the amplitude and temporal profile of the MA measured on the scalp compared with that on the reference layer. For movements, such as a head shake, containing a rotational motion about a component of the magnetic field an additional homopolar voltage will be created due to the addition of relative motion of elements of the volume conductor (skin and brain).

As a result of the multiple contributions to the MA induced by a head shake, correlation of the signals on the two layers is considerably reduced and simple fitting of reference signals (either single or multiple channel) to the scalp signals is unlikely to be able to remove this artefact component completely due to the difference in temporal profile. We provide evidence and theory suggesting that this discrepancy is caused by the movement of the brain relative to the skin which cannot be captured by motion tracking devices on the surface of the EEG cap, as these only move with the skin. Given the relative success of MPT markers for the correction of the MA it may be possible to use these to track the motion of both the skull and scalp, combining the motion tracking techniques performed by Maziero et al. (2016) with those of Le Van et al. (LeVan et al., 2013) to provide optimal MA correction. However, this will provide new technical challenges to get the line of sight between both MPT markers and the camera, although MRI- based work has shown that it is feasible to track 2 MPT markers during MRI data acquisition (Singh et al., 2015). An alternative method which may minimise the homopolar contribution would be to reduce the amount of padding around the head, thus reducing the resistance the skin experiences to movement. However, it is likely that at the point of contact (the back of the head) the skin and skull will never produce rigid body movement.

Given the complexities of correcting the MA highlighted in this work it may be tempting to still simply reject periods of data where a MA is generated, as originally proposed (Bonmassar et al., 2002; Allen et al., 1998). However, making decisions about which data to reject is difficult, as the amplitude of the MA is dependent on the rate of change of head position (i.e. velocity) rather than total displacement. Therefore, it would not possible to discard EEG data purely based on the peak-to-peak amplitude of a movement, the time taken for this movement must also be considered. For accurate characterisation of these movement related parameters a high sampling rate of head motion, like that provided by the MPT, is required. Furthermore, the amplitude of MA which will be problematic to the EEG data quality is likely to depend on the brain signals of interest as well as the frequency band they reside in, as, for example, alpha oscillations are much larger in amplitude than gamma oscillations, thus are less likely to be swamped by small MAs. Therefore it is not possible to provide prescriptive guidance on when data should be excluded due to MA, highlighting the importance of further work in this area to provide robust methods to remove all forms of MA.

## Conclusions

Comparison of MPT measurements with EEG recordings during controlled movements of the head show that the MA is not as simple as previously thought, with complex, non-rigid body elements of the motion contributing significantly to the measured artefact voltages. Moreover, head-shake movements, could produce a homopolar generator effect as a result of rotational motion of the skull and brain relative to the scalp. While this makes the correction of MAs using reference signals more challenging, provided that the driving motion can be linearly related to the MA recorded on the scalp the MA may be amenable to fitting and subtracting of the reference signals.
