# Supplementary material for: Exploring the origins of EEG motion artefacts during simultaneous fMRI acquisition: Implications for motion artefact correction
Source: Neuroimage. 2018 Jun;173:188–98. doi: 10.1016/j.neuroimage.2018.02.034 (PMC5929889; doi:10.1016/j.neuroimage.2018.02.034)
Supplement: Material [file mmc1.docx]

**Supplementary Information**

**Results**

***Figure S1****: Schematic showing placement of MPT markers on the head and EEG cap for* ***A:*** *the standard EEG cap and* ***B****: the RLAS EEG cap.*

**Figure S2:** Time-courses of MPT data taken from a representative subject. MPT markers were fixed to a bitebar, the nasion and the centre of the forehead. Panel **A** shows the change in position of individual markers relative to the camera frame and panel **B** shows the change in position between pairs of markers. Types of movement are separated column-wise showing: **(i)** a rest period, **(ii)** a continuous head-nod motion period and **(iii)** a period of head-shake. Note, a 2/1 mm offset between time-course baselines is employed to aid visualisation in panels **A** and **B,** respectively.

***Figure S3****: Flat-maps showing the RMS values of the EEG data collected on subjects wearing standard 32 channel EEG caps. The values shown are calculated as an average across all three subjects. Map* ***A*** *shows the results for a nod motion. Map* ***B*** *displays the results from a shake motion.*

**Table S1:** Root-Mean-Square (RMS) measures of motion data in mm for markers attached to four healthy subjects. Mean and standard deviation calculated across subjects. Markers were affixed to a bite-bar, the nasion and the forehead at the site of Fpz. **A**: Shows the RMS of high-pass filtered motion data of individual markers relative to the camera for motions “Rest”, “Nod” and “Shake”. **B**: Shows the RMS of the change of position between pairs of markers for the three types of movement.

**Table S2:** Root-Mean-Square (RMS) measures of motion data in mm for three markers all attached to the surface of a head-shaped gel phantom. **A**: Shows the RMS of high-pass filtered motion data of individual markers (Mk1 to Mk3) relative to the camera for motions “Rest”, “Nod” and “Shake”. **B**: Shows the RMS of the change of position between pairs of markers for the three types of movement.

**Discussion**

This homopolar generator model can also explain the difference in the artefact voltages induced on the scalp and reference layer for head nod and head shake. In the case of head shaking the location of the reference electrode is on the axis of rotation whilst the other electrodes are sited some distance from this point, such that large voltages are induced at their locations. Whereas for a perfect nod motion, the rotation is about an axis perpendicular to the magnetic field. This means that there will not be a radial component to the electric field meaning that no potential difference will be created between the reference electrode and another channel by the homopolar mechanism. Therefore no homopolar contribution is expected for a nodding motion. This suggestion of a homopolar contribution to the artefact is reflected by the results shown in Table S3, where the phantom nod and shake motions (where there is no skull-skin movement) and the subject’s nod motion (where no homopolar contribution is expected) all show a similar RMS value for the potential difference between the scalp and reference layer reference electrodes (with a ground electrode located closer to the equator of the head) which are relatively small. However, the shake motion for a subject shows a large difference between the recordings on the reference electrode pair. For a perfect conductive sphere of radius 8.75 cm rotating at an angular rate of 0.01 rad/s about a field of 3T, the potential difference between the pole and the equator can be calculated to be approximately 115 μV, which is not dissimilar to values reported in Table S3 for a subject executing a shake motion.

**Table S3**: Average over electrode pairs of the RMS potential difference between reference layer and scalp electrodes at the reference electrode location for three phantom and subject recordings for nod and shake. A common ground was employed for both layers.
